# Weather stressors correlate with *Escherichia coli* and *Salmonella enterica* persister formation rates in the phyllosphere: a mathematical modeling study

**DOI:** 10.1038/s43705-022-00170-z

**Published:** 2022-09-27

**Authors:** Maria T. Brandl, Renata Ivanek, Nerion Zekaj, Alexandra Belias, Martin Wiedmann, Trevor V. Suslow, Ana Allende, Daniel S. Munther

**Affiliations:** 1 Produce Safety and Microbiology Research Unit, US Department of Agriculture, Agricultural Research Service, Albany, CA 94710 USA; 2 Department of Population Medicine and Diagnostic Sciences, College of Veterinary Medicine, Cornell University, Ithaca, NY 14853 USA; 3 Department of Mathematics and Statistics, Cleveland State University, Cleveland, OH 44115 USA; 4 Department of Food Science, Cornell University, Ithaca, NY 14853 USA; 5 Department of Plant Sciences, University of California, Davis, CA 95616 USA; 6 Research Group of Microbiology and Quality of Fruit and Vegetables, Food Science and Technology Department, CEBAS-CSIS, Campus Universitario de Espinardo, Murcia, E-30100 Spain

**Keywords:** Applied microbiology, Microbial ecology

## Abstract

Enteric pathogens can enter a persister state in which they survive exposure to antibiotics and physicochemical stresses. Subpopulations of such phenotypic dormant variants have been detected in vivo and in planta in the laboratory, but their formation in the natural environment remains largely unexplored. We applied a mathematical model predicting the switch rate to persister cell in the phyllosphere to identify weather-related stressors associated with *E. coli* and *S. enterica* persister formation on plants based on their population dynamics in published field studies from the USA and Spain. Model outputs accurately depicted the bi-phasic decay of bacterial population sizes measured in the lettuce and spinach phyllosphere in these studies. Predicted *E. coli* persister switch rate on leaves was positively and negatively correlated with solar radiation intensity and wind velocity, respectively. Likewise, predicted *S. enterica* persister switch rate correlated positively with solar radiation intensity; however, a negative correlation was observed with air temperature, relative humidity, and dew point, factors involved in water deposition onto the phylloplane. These findings suggest that specific environmental factors may enrich for dormant bacterial cells on plants. Our model quantifiably links persister cell subpopulations in the plant habitat with broader physical conditions, spanning processes at different granular scales.

## Introduction

Bacterial immigrants in the phyllosphere encounter heterogeneous physicochemical conditions dominated by solar radiation, water scarcity, temperature fluctuations, nutrient limitation, and antimicrobials that determine their fate in the plant environment [1–4]. Populations of human enteric pathogens inoculated onto crops in the field commonly undergo bi-phasic decay [5–11]. This trend is characterized by a large, rapid population decline after inoculation, followed by the survival of small subpopulations that remain relatively stable over time. In light of the recurrent outbreaks of enteric illness linked to fruit and vegetables [12–14], these few surviving cells are of high interest since they may represent subpopulations that serve as a reservoir for further amplification of the pathogen upon resumption of growth-permissive conditions on plants in the field or after harvest. Given the low infectious dose of pathogens such as Shiga toxin-producing *E. coli* (STEC) [15], initiation or resumption of pathogen multiplication on edible crops increases the probability of foodborne disease and the risk to public health.

The spatiotemporal heterogeneity of plant surfaces implies that clonal bacterial plant colonists may form subpopulations with distinct physiologies. Multiple bacterial pathways have been investigated at the single bacterial cell level on leaf surfaces and have been shown to vary greatly in activity among subpopulations of the inoculated reporter strain [1, 4, 16]. Given this heterogeneity, investigating the behavior of bacteria at the scale of single cells is critical to a comprehensive understanding of their ecology in the phytosphere. During bean leaf colonization by a *Pantoea agglomerans* bioreporter of cell replication, 3% of the viable cells did not divide once while the overall population of the bioreporter increased tenfold [17], suggesting the presence of a small subpopulation with distinct low metabolic activity. The role of phenotypic heterogeneity within clonal bacterial populations exposed to stressors has come to light through the discovery of persister subpopulations of human pathogens that survive antibiotic treatment without harboring mutations or genetic determinants imparting antibiotic resistance [18]. Mediated by metabolic dormancy likely resulting from ribosome inactivation [19], the persister state is viewed as a strategy to ensure the survival of a few in a population under stress. Current evidence supports that in addition to stochasticity, mechanistic processes induce persister cell formation in response to environmental cues via defined stress response pathways [20, 21]. Such cues include nutritional, acidic, oxidative, osmotic, and antimicrobials stresses. Importantly, although persister and viable-but-nonculturable (VBNC) cells may be part of a dormancy continuum, the persister state is transient and thus, cells may reverse from dormancy to a replicating state that allows for their recovery on culture media without the prolonged and specific resuscitation conditions required for culturing of VBNC cells [22].

We have recently reported the presence of *E. coli* O157:H7 (EcO157) persister cells after inoculation of the pathogen into irrigation water and spinach leaf wash water [23] and onto the lettuce phyllosphere of plants grown in the laboratory [24]. In the latter study, the pathogen population underwent bi-phasic decline on the leaves of lettuce plants exposed to dry conditions. Additionally, the persister cell fraction of the EcO157 population was greater under these dry conditions than during plant conditions that effected pathogen population growth or stability on the leaves. We further developed a mathematical model based on our laboratory persister data to describe EcO157 switch rates from nonpersister cells to persister cells on leaves of lettuce plants left to dry after inoculation. Applying our model equations from the decay regime on laboratory-inoculated plants, we estimated model parameters for four previously published field studies (in 2010–2013) of EcO157 survival on lettuce and obtained switch rates similar to those observed on lettuce in our laboratory study [24]. Hence, the model accurately described the survival rates of EcO157 in the lettuce phyllosphere in several field studies, all of which had shown bi-phasic survival of the pathogen [24].

The contamination of edible crops with enteric pathogens has become a major threat to public health and to the horticulture industry. Contaminated fruit and vegetables have caused numerous outbreaks of foodborne illness worldwide [12, 13, 25, 26]. In particular, leafy greens have been associated with large epidemics of STEC and *S. enterica* infections in the USA, Canada, and Europe [14, 27, 28]. Our understanding of the ecology of human enteric pathogens on plants in relation to environmental stressors dictating their behavior and physiology *in planta* in the field is essential to the development of strategies to enhance crop safety. In this study, we used mathematical modeling and data published by Belias et al. in 2020 on the survival of *E. coli* and *S. enterica* inoculated onto spinach and lettuce plants in field trials in California, New York, and Spain under known weather conditions [9] in order to 1) estimate the switch rate of the pathogens to persister cells on these crops in the field; and 2) explore the relationship between weather factors and the rate of persister cell formation in the phyllosphere.

## Materials and methods

### Case study

The experimental setup for the field studies that provided the bacterial population and weather data used here was previously described by Belias et al. [9]. Briefly, baby spinach and lettuce plants were spray-inoculated with *E. coli* and *S. enterica* (*Salmonella*) onto field plots established in Davis, CA (University of California, Plant Sciences Field Research Facility); Freeville, NY (Homer C. Thompson Research Farm, Cornell University); and Murcia, Spain (La Matanza Research Farm). The spinach and lettuce varieties were selected based on their suitability for baby leaf production: lettuce var. Tamarindo, and spinach var. Acadia F1 and Seaside F1. Four replicate trials at different times of the regional growing season were carried out per location. The plants were spray-inoculated with a 10^4^ CFU/mL cocktail of rifampin-resistant strains of commensal *E. coli* and attenuated *S. enterica* serovar Typhimurium (*Salmonella*), and samples were collected for bacterial cell quantification by plate counts on selective and differential media at 0, 4, 8, 24, 48, 72 and 96 h post-inoculation. Concurrent with leaf sample collection, weather variables (temperature, relative humidity (RH), solar radiation intensity, and wind velocity) were recorded hourly for the respective field locations. The hourly dew point (DP) was calculated as a function of both the hourly temperature and RH.

### Model for persister formation on plants

Mathematical modeling to characterize the switch rate from a non-persister bacterial cell (hereafter termed “normal cell”) to a persister cell in the phyllosphere under laboratory conditions was performed as described in our previously published study [24]. Briefly, persister cell fractions were quantified in culturable EcO157 populations after inoculation onto young lettuce plants cultivated in plant growth chambers. Persister cells recovered from the lettuce phyllosphere were identified using the antibiotic lysing method [23]. The greatest persister fraction in the EcO157 population on lettuce in our laboratory investigation above was observed during population decline on leaf surfaces of plants left to dry after inoculation. Using mathematical modeling, we calculated the switch rate from an EcO157 normal to persister cell on dry lettuce plants based on these data [24]. Importantly, our laboratory conditions mimicked inoculation conditions in which *E. coli* arrived via water on leaves, the surfaces of which progressively dried like under prevailing weather conditions in the field.

Based on the main dynamic observed in the field study data [9] and building on our previous study [24], we assumed that the total enteric pathogen population is composed of (i) non-persister (normal) cells consisting of two sub-populations, characterized by fast (*n*_1_) (CFU/100g) and slow (*n*_2_) (CFU/100g) decay, and (ii) the persister population, leading to the following model from Munther et al. [24]:

1a $$\frac{{dn_1}}{{dt}} = - \theta _{n_1}n_1 - \alpha _dn_1 + \beta _d\left( {1 - \sigma } \right)\hat p,$$

1b $$\frac{{dn_2}}{{dt}} = - \theta _{n_2}n_2 - \alpha _dn_2 + \beta _d\sigma \hat p,$$

1c $$\frac{{d\hat p}}{{dt}} = - \mu _{\hat p}\hat p - \beta _d\hat p + \alpha _d\left( {n_1 + n_2} \right),$$

1d $$n_1\left( 0 \right) = n_{10},n_2\left( 0 \right) = n_{20},\, \hat p\left( 0 \right) = \widehat {p_0},$$

where $\theta _{n_i}$(1/*h*) is the death rate of the normal cells (subscript *i* = 1 for fast and *i* = 2 for slow), $\hat p$ (CFU/100 g) represents the persister cell population at time *t* (h), $\mu _{\hat p}$ (1/*h*) reflects the persister population inactivation rate, *α*_*d*_ (1/*h*) is the switch rate from normal to persister state, *β*_*d*_ (1/*h*) is the switch rate from persister to the normal state, and *σ* ∈ (0,1) is a constant, describing the fraction of persister cells switching back to the normal, slowly decaying state. Equation (1a) and (1b) reflect the assumption that times between switching states are exponentially distributed, using the expected values $\frac{1}{{\alpha _d}}$ (h) and $\frac{1}{{\beta _d}}$ (h) of the respective distributions.

Lacking data for potential persister populations from the field trials, *we assumed the persister population is a fraction 1* *>* *k* *>* *0 of the tail population*, as observed in Munther et al. [24]. Regarding the model above, this implies that $\hat p \approx kn_2$ for $t \ge t^ \ast$, where $t^ \ast \approx \frac{1}{{\theta _{n_1}}}$ (the time scale of survival for the fast-decaying population (*n*_1_)). In accord with bi-phasic decay, for $t \ge t^ \ast$, the main dynamics for slow decaying population (*n*_2_) is dictated by $- \theta _{n_2}n_2$ in Eq. (1b). This suggests that the effective switch rates from *n*_2_ to $\hat p$ and from $\hat p$ back to *n*_2_ balance, so that $\beta _d\sigma \hat p \approx \alpha _dn_2$ in Eq. (1b). Following these ideas, we simplified the model in Eq. (1a)–(1d) to:

2a $$\frac{{dn_1}}{{dt}} = - \theta _{n_1}n_1 - \alpha _dn_1,$$

2b $$\frac{{dn_2}}{{dt}} = - \theta _{n_2}n_2,$$

2c $$\frac{{d\hat p}}{{dt}} = - \theta _{\hat p}\hat p + \alpha _dn_1,$$

2d $$n_1\left( 0 \right) = n_{10},n_2\left( 0 \right) = n_{20},\, \hat p\left( 0 \right) = \widehat {p_0},$$

where we ignored $\beta _d\left( {1 - \sigma } \right)\hat p$ in (1a) since the decay rate ($\theta _{n_1}$) dominates. Also, by setting $\theta _{\hat p} = \mu _{\hat p} + \beta _d(1 - \sigma )$, and using $\beta _d\sigma \hat p \approx \alpha _dn_2$, we obtained Eq. (2c). Furthermore, because $\hat p \approx kn_2$ for $t \ge t^ \ast$, $\theta _{\hat p} \approx$ $\theta _{n_2}$.

In particular, the assumption that $\hat p \approx kn_2$ for $t \ge t^ \ast$ characterizes the switch rate from normal to persister cells, *α*_*d*_, as $\alpha _d \approx k\alpha$, where *α* is a *hypothetical* switch rate assuming that the population is composed only of fast decaying normal cells (*n*_1_) and a *hypothetical* persister cell population (*p*). In this case, the hypothetical population *p* starts small at $\widehat {p_0}$, initially increases due to switching from population *n*_1_ and then slowly decays as the *n*_1_ population is effectively inactivated (i.e., the tail of the total population is comprised entirely of *p*). From this perspective we utilized the following equations:

3a $$\frac{{dn_1}}{{dt}} = - \theta _{n_1}n_1 - \alpha n_1,$$

3b $$\frac{{dp}}{{dt}} = \alpha n_1 - \theta _pp.$$

3c $$n_1\left( 0 \right) = n_0,\, p\left( 0 \right) = \widehat {p_0},$$

For mathematical justification regarding the relationship $\alpha _d \approx k\alpha$, please see the appendix (Supplementary Information).

The utility of the relationship $\alpha _d \approx k\alpha$, is twofold. First, we used model fitting (Eqs. (3a)–(3c)) to determine *α* from the respective field study data [9]. Note that using Eqs. (3a)–(3c), we actually fit for $\theta _{n_1}$, *θ*_*p*_, and *α* using the field study data [9]. Please reference the “model fitting procedure” section as well as the appendix for details concerning the unique determination of the aforementioned parameters, i.e., the practical identifiability of these parameters, and justification regarding the legitimacy of measured tail populations relative to the respective field trial data [9]. Second, because we wanted to examine Spearman’s correlations (corr) between *α*_*d*_ and various weather factors, given a particular weather factor $\vec w$ across trials $i = 1, \ldots ,n$, let *k* be the maximum persister fraction (of the tail) across these *n* trials, that is, for each *i*, we have $\alpha _{d_i} \approx k_i\alpha _i$, so $\alpha _{d_i} \lesssim k\alpha _i$. Thus *kα*_*i*_ represents the maximum persister switch rate for each trial *i*, and since corr($k\vec \alpha ,\vec w$) =corr($\vec \alpha ,\vec w$), we conducted the correlation analysis with the fitted *α* values in lieu of the actual persister switch rate *α*_*d*_.

The assumptions behind our approach are summarized below:

A. The tails of pathogen populations surviving on plants in the field study [9] are comprised of some fraction *k* ∈(0,1) of persister cells since their decay rate is quite small and they remain culturable.
B. Because $\alpha _d \approx k\alpha$, we hereafter utilize *α* from model (3a)–(3c) as the representative persister switch rate.
C. Given that the experimental context [24] for modeling persister switching occurred during population decline, we only employed trials from Belias et al. [9] that exhibited bi-phasic decay. Namely, we did not include trials in which significant bacterial growth was observed at the time scale of successive data points (the time scale in the field study is on the order of 4–16 h for the 1st day and then 24 h thereafter.)
D. The switch rate from normal to persister cell is on average a monotonic function of some measure of environmental stress.

Based on assumptions A–D above, we applied the model (3a)–(3c) to published pathogen population size and weather data from four replicate trials in Spain, two in California, and one in NY [9]. More specifically, we fit model (3a)–(3c) to the respective population data in order to:

1. determine values for the maximum switch rate *α* relative to the produce/bacteria type at the field scale,
2. describe the correlative relationship between *α* and weather factors in the respective field trials.

### Model fitting procedure

In model (3a)–(3c) above, we supposed dp/dt_t = 0_ > 0, i.e., we assumed that bacteria experience stress from the change in conditions from culture growth and inoculum suspension preparation to those on the plant surface and therefore, that persister formation increases in the phyllosphere immediately following inoculation. The report that EcO157 persister formation increases as early as 1 h after inoculation into leaf wash water [23], which could be considered as a proxy for the average oligotrophic environment that bacterial cells experience after spray inoculation onto leaves or through irrigation in the field, supports this assumption. To avoid identifiability issues between the initial persister population $\widehat {p_0}$ and *α* regarding the model fits above, we assumed that $\widehat {p_0}$= 1 ($\widehat {p_0}$ = 0 gives the same results). Thus, the initial persister population at inoculation is at its lowest, an assumption supported by Munther et al. [24], who observed an average fraction of EcO157 persisters of 0.0043% in the inoculum population. This imparts the largest possible switch rate, *α*, onto the population, corresponding to the largest and hence most conservative food safety risk.

Let *y*_*k*_ (CFU/100 g of produce) be the average bacteria population measurement at time *t*_*k*_ (h) and let *P*_*k,X*_ (CFU/100 g of produce) represent the model prediction (total population) at time *t*_*k*_ relative to the parameter vector $X = [ {\theta _{n_1} \,\ \theta_p \,\ \alpha } ]^T$. Following Eqs. (3a) and (3b), this means that ${{{{{{{\mathrm{P}}}}}}}}_{k,X} = n_1\left( {t_k,X} \right) + p(t_k,X)$. Since the population data spans multiple orders of magnitude, we calculated the residuals as $e_{k,X} = \log _{10}y_k - \log _{10}P_{k,X}$. To determine the optimal model fit (see the appendix for details regarding *a priori* bounds on parameter ranges), we utilized the *fminsearch* function in MATLAB (MATLAB 2020b, The MathWorks, Inc., Natick, Massachusetts, United States) to determine the parameter vector *X* that minimizes the 2-norm of the following function *F*:

$$| | F\left( X \right) | |_2 = \left( {\mathop {\sum }\limits_k e_{k,X}^2} \right)^{\frac{1}{2}}$$

### Correlation analysis

**Fig. 1:**
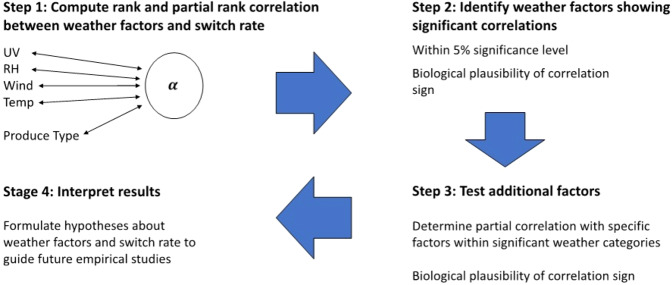
Logical flow diagram for statistical analysis. Factors in Step 1: UV (average ultraviolet radiation intensity), RH (average air relative humidity), Wind (average wind speed), and Temp (average air temperature). All weather data used in the statistical analysis were obtained over 8 h post-inoculation of *E. coli* and *Salmonella* onto lettuce and spinach leaves in the field.

To provide a statistical foundation from which to relate the switch rate *α* and measured weather factors, we utilized Spearman and partial Spearman correlation. First, we calculated the Spearman correlation coefficients between *α* and each of the respective factors: 8-h average of temperature, RH, solar radiation, wind speed post-inoculation, and then we calculated the partial Spearman correlation coefficients for each respective weather factor, while controlling for the other three factors and simultaneously controlling for produce type (using lettuce =1 and spinach =0) (For details regarding why 8-h weather variables were used, see the “model fitting” subsection of the results.) The correlation coefficients were determined using the *corr* and *partialcorr* functions in MATLAB 2020b (The MathWorks, Inc., Natick, MA, USA). Considering the significant association of *Salmonella α* with RH and temperature, we also examined the correlation between *α* and dew point. Figure 1 presents a logical flow of the statistical analysis. Partial correlations with a *P* value of less than 0.05 were deemed significant. If the 8-h average of a weather factor exhibited a significant correlation with the switch rate, the 8-h minimum and range of the weather factor were also tested.

## Results and discussion

### Model fitting

**Fig. 2:**
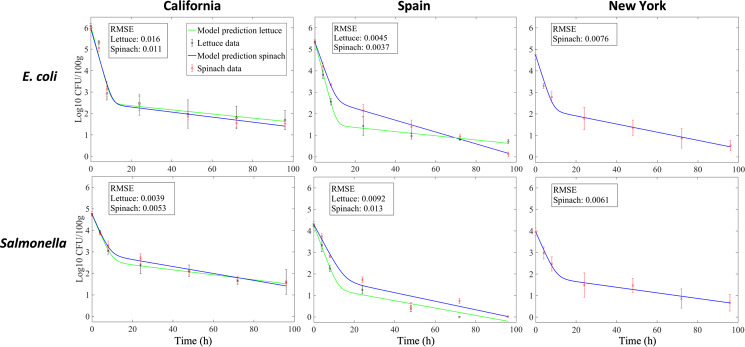
Population size dynamics of *E. coli* (upper panel) and *Salmonella* (lower panel) in the lettuce and spinach phyllosphere in field trials in California, Spain, and New York, and persister model fits for each crop (green line, lettuce; blue line, spinach). Sampling times were 0, 4, 8, 24, 48, 72, and 96 h post-inoculation. Each datum point represents the average population size and standard deviation from four subplots per trial from two, four, and one field trial(s) in California, Spain, and New York, respectively, as detailed in Materials and Methods. For details regarding the frequency of samples above the limit of detection (6 CFU/100g) for the tail populations refer to the “Details for parameter fitting” section in the appendix. The root mean square error (RMSE) from the optimal fits for population dynamics on each crop is provided as log10 CFU/100g in each figure.

Model (3a)–(3c) describes the dynamics of bacterial persister formation after a population of cells arrive in an aqueous carrier (irrigation or inoculation solution) on leaf surfaces in the field and their physical and chemical environment subsequently changes due to gradually drying conditions in the phyllosphere. Figure 2 shows data obtained at all three locations, Spain, New York, and California in the field study by Belias et al. [9] and the model output resulting from fitting the model to these data. A rapid decline of *E. coli* and *Salmonella* population sizes was observed on both spinach and lettuce plants within 10–24 h post-inoculation. This early steep decline has been observed in several similar field studies about enteric pathogen survival on leafy vegetables [5–7, 10, 29, 30] as well as after inoculation of successful plant colonists such as *Pseudomonas syringae* and *Pantoea agglomerans* onto various plant species in the field [31, 32]. Whereas the bacterial epiphytes in the latter studies had the ability to multiply in the leaf habitat following this early decline, surviving enteric pathogens generally make up a small, and stable or slowly decaying population on the aerial surfaces of plants, similarly to trends shown in Fig. 2 and in other field studies cited above.

Following procedures described above, we tested the fit of model (3a)–(3c) with the *E. coli* and *Salmonella* population dynamics observed in each of the field trials in California, New York and Spain (averaged across four field subplots per time point for each of the respective field trials, thus satisfying assumption C above). Figure 2 illustrates the average population sizes of each pathogen at each sampling time on lettuce and spinach leaves, demonstrating that the model captures the bi-phasic decay pattern. More specifically, the root mean square error from the optimal fits for *E. coli* dynamics on lettuce and spinach ranged from 0.0037 to 0.016 log10 CFU/100g and from 0.0039 to 0.013 log10 CFU/100g for *Salmonella* (Fig. 2) (Values of the parameters $\theta _{n_1}$(fast decay), *θ*_*p*_ (slow decay), and *α* (switch rate) from the fitting results illustrated in Fig. 2, are listed in Table S1 in the appendix.)

In order to obtain maximum information regarding the switch rate (α), we fit model (3a)–(3c) to field subplot data (rather than averaging across all subplots in the same field) for each respective trial at each location. From the results of the model fitting at the subplot level, $T_{max}$ ≲ 10 h, that is, the persister population is predicted to reach a maximum within 10 h after the pathogen cells arrived on the leaves. Relative to this time scale, we only used weather information from the first 8 h post-inoculation in our analyses. From a mechanistic point of view this is illustrated in model Eq. (2c), as the switching mechanism (from normal to persister cells) dominates the persister population dynamics until the fast-decaying population becomes quite small, on a time scale proportional to $1/\theta _{n_1}$. Notice that averaging across the fits from all trials, $1/\theta _{n_1} \approx$ 16 h, which is relatively close to *T*_*max*_ computed above.

### *E. coli α* and weather parameters

Regarding direct correlations, the *E. coli* switch rate *α* was significantly correlated with the average 8-h temperature, RH, and solar radiation (Table 1). Partial rank correlation results (Table 1) indicated that only solar radiation and wind speed showed a significant correlation with the switch rate *α* while controlling for the other factors. Controlling for produce type in the partial correlation analyses did not have a substantial effect on the results. Thus, plant species was not a significant factor in the correlation between switch rate and stressor. This lack of difference between spinach and lettuce may be attributable to inoculation of the enteric pathogens onto the plants at an early growth stage (baby leaf) in the field studies by Belias et al. [9]. At this stage, both plant species would have young leaf tissue and an open canopy structure that may mediate overall similar physicochemical conditions experienced by bacterial cells on the phylloplane with respect to the weather stressors that we investigated. Indeed, Fig. 2 reveals similar enteric pathogen survival curves in both plants. It is probable that at maturity, plant species and in particular lettuce type would affect the results of a correlation analysis since the plant morphology varies greatly at that stage and this may generate differences in stresses from weather factors impacting bacterial cells on the leaf surface.

**Table 1:** Direct and partial Spearman correlation* of *E. coli* persister switch rate *α* with an average of 8-h weather factors.

	Temperature	RH	Solar Radiation	Wind speed
*ρ*	0.44	0.38	0.59	0.21
*P* value	0.0081	0.025	0.002	0.22
*δ*	0.28	0.067	0.51	−0.36
*P* value	0.11	0.72	0.003	0.048

*n = 35; *ρ* indicates direct Spearman correlation; *δ* symbolizes partial Spearman correlation. Partial correlation between a given weather factor (in row 1) and *α* is determined by controlling for the other three weather factors in row 1 as well as controlling for produce type.

Table 1 indicates several important points. First, these results justify the importance of utilizing partial correlation to uncover interdependencies that may exist between multiple weather factors and the switch rate *α*. From this perspective, we placed more weight on the partial correlation results (rather than direct correlation), using these to provide statistical evidence in relation to established biological insight regarding the aforementioned assumption (D) and observed physical relationships between various weather factors. Contrasting results in Table 1, when controlling for the other respective weather factors, the correlations between the factors temperature and RH, and *α* did not remain statistically significant.

One possible explanation for temperature is its significant correlation with solar radiation and therefore temperature serves as a proxy for increased solar exposure. This conjecture is supported by the strength of the correlation between temperature and average solar radiation while controlling for RH (partial Spearman *δ* = 0.77, *P* = 9e-8). It is unclear why RH in the phyllosphere would not be correlated with the persister switch rate of *E. coli*. RH does not imply the presence or absence of free water on the phylloplane, since this would also depend on dew point; hence RH is not a direct measure of desiccation stress on bacterial cells. However, it is also plausible that desiccation is not an inducer of the persister state in *E. coli* since this has not been documented in the literature to date. Partial Spearman correlation was tested also between switch rate and the factors “temperature range”, “RH range”, and “dew point”, and results failed to show a significant association (data not shown). Therefore, contrary to what we describe below in *Salmonella*, none of the three tested weather factors that would contribute to the presence or absence of free water on the phylloplane showed any correlation with switch rate in *E. coli* in our study.

### Solar radiation

Solar radiation showed a significantly moderate correlation with *α*, both directly and when controlling for the other weather factors and produce type. This positive correlation suggests that *E. coli* cells on lettuce and spinach leaves increasingly switch to the persister state with increasing solar radiation. The SOS response in *E. coli*, a ubiquitous bacterial strategy to repair DNA damaged by various stresses, including UV radiation, has previously been reported to induce persister cell formation [33]. As epiphytes, plant-associated bacteria benefit from DNA repair mechanisms such as those involving the SOS regulon, as well as pigmentation and spatial avoidance at particular microsites on the leaves, during colonization of leaf surfaces under solar radiation [34–36]. It was shown that protection of peanuts plants from UV-B lowered the relative abundance of UV-tolerant bacterial species in the phyllosphere community, suggesting that exposure to solar radiation enriched for UV-tolerant species [37]. Furthermore, *Pseudomonas* population sizes on baby spinach in the field correlated negatively with solar radiation providing further evidence that exposure to high radiation intensity imposes stress on bacterial cells [38]. Reduction of solar radiation exposure by shading plants [39] or inoculating the abaxial side of leaves [6] in the field improved *E. coli* O157:H7 survival in the spinach and lettuce phyllosphere, respectively; however, a direct correlation between population sizes and radiation intensity was not established. Overall, this suggests that UV exposure of bacterial cells in the phyllosphere may select for subpopulations composed at least partly of persister cells.

### Wind velocity

The weak but significant negative association between wind velocity and α based on partial Spearman correlation in Table 1 is noteworthy since it implies that high wind speed inhibits the persister cell switch rate in *E. coli* on plant surfaces. High wind velocity, which disrupts the boundary layer of leaf surfaces [40] lowers the leaf temperature under conditions such as high solar radiation [41]. It may therefore alleviate stress on certain bacterial cells in the phylloplane and lower the formation rate of *E. coli* persisters. It is possible also that the movement of leaves in the canopy under high winds results in a shading effect and thus shorter exposure of the bacterial cells to UV radiation at any given time. The role of wind speed in the survival of bacterial residents on plants has not been investigated extensively. Average population sizes of *Enterobacteriaceae* and *Pseudomonas* spp. on spinach leaves in the field were reported to be negatively correlated with wind velocity [38]. Thus, while the population as a whole may be negatively impacted under high wind, a small subpopulation may experience a given stress alleviation mediated by this high wind velocity. One could hypothesize that the deleterious or beneficial effect of wind on bacteria in the phylloshere results indirectly from other environmental factors related to the disturbance of the laminar layer, where bacterial cells are located.

### *Salmonellaα* and weather parameters

The strongest correlations between *α* and measured weather factors were with RH, followed by solar radiation and temperature (Table 2). Comparing the results in Table 2 again illustrates the suitability of using partial Spearman correlation since direct correlation masks significant correlations between *α* and temperature and solar radiation.

**Table 2:** Direct and partial Spearman correlation* of *Salmonella* persister switch rate *α* with average of 8-h weather factors.

	Temperature	RH	Solar Radiation	Wind speed
*ρ*	−0.01	−0.64	0.24	0.10
*P* value	0.60	0.0002	0.21	0.60
*δ*	−0.52	−0.81	0.74	−0.033
*P* value	0.01	6.8e-7	1.8e-5	0.87

*n = 30; *ρ* indicates direct Spearman correlation; *δ* symbolizes partial Spearman correlation. Partial correlation between a given weather factor (in row 1) and *α* is determined by controlling for the other 3 weather factors in row 1 as well as controlling for produce type.

### Solar radiation

As observed also for *E. coli* switch rates (Table 1), solar radiation is positively correlated with the persister cell switch rate in *Salmonella*. Induction of persister cell formation by the SOS response has not been extensively investigated in *Salmonella* as in *E. coli*. However, the high similarity in their response network to DNA repair [42] would support a similar positive correlation of *α* in *Salmonella* with exposure to solar radiation in the phyllosphere.

### RH and temperature

Contrary to our observations in *E. coli*, *α* is highly associated with RH in *Salmonella*. The negative correlation suggests that the persister formation rate decreases as RH in the lettuce and spinach environment increases. A relative humidity of 80% enhanced the survival of *Salmonella*, compared with 60%, in overall decaying populations on young lettuce plants in growth chamber studies [30], supporting this interpretation. Building on the significant results in Table 2, we calculated the partial correlations between *α* and minimum values and range for temperature, dew point, and RH, while controlling for the remaining three weather categories as well as produce type. Table 3 illustrates that each of these specific factors was significantly correlated with *α*. Minimum temperature and RH show a negative *δ* (partial Spearman correlation) value, likely due to the stress imposed onto *Salmonella* cells by cold temperature and dry air. In addition, the range in temperature, dew point, and RH over the 8-h period after inoculation used in our model was positively associated with the persister switch rate. Remarkably, it was also demonstrated by Lopez-Galvez et al. that large fluctuations in RH every 12 h negated the benefit of constant high RH in *Salmonella* survival on lettuce [30]. This observation and the results we describe herein support current thinking that large fluctuations in physicochemical conditions in the plant habitat impose additional stress onto bacterial residents [43]. It is unclear why the switch rate to persister cells in *E. coli* was not similarly affected by temperature and RH range. However, as neither the minimum nor the average values of these parameters were significantly associated with *E. coli α* based on partial Spearman correlation (Table 1), this may corroborate that temperature and RH were not significant triggers of the persister state in *E. coli* on plants in our study.

**Table 3:** Partial Spearman correlation* of *Salmonella* persister switch rate *α* with specific 8-h weather factors.

	*δ*	*P* value
Minimum Temp	−0.58	0.002
Temp Range	0.57	0.003
Minimum RH	−0.82	3.2e-7
RH Range	0.83	1.8e-7
Average dew point	−0.66	1.7e-4
Dew point range	0.86	6.0e-9

*n = 30; For partial correlation between Minimum Temp/Temp Range and *α*, solar radiation, RH, and wind speed were controlled for as well as produce type. For partial correlation between Minimum RH/ RH range and *α*, solar radiation, Temp, wind speed, produce type were controlled for. Partial correlations between the respective dew point factors and the switch rate *α*, controlled for solar radiation, wind speed and produce type.

Whereas the direction of the correlation between *α* and solar radiation and RH makes intuitive sense since an increase in their magnitude would likely translate to an increase and decrease in cellular stress, respectively, the negative association of average temperature with *Salmonella α* (Table 2) requires more careful interpretation. The partial Spearman correlation between temperature and dewpoint (while controlling for RH) for *Salmonella* trials is very high (*δ* = 0.94, *P* value = 9.2e-16) (data not shown). Considering the monotonic relationship between temperature and dew point (given a fixed RH) [44], we can explain the negative correlation between temperature and *α* based on the strong negative correlation between dew point and *α* (Table 3). One could then surmise that increasing temperatures accompanied by leaf wetness support increasing multiplication of *Salmonella* in the phyllosphere, as demonstrated previously [45], and hence inhibit persister formation or cause a reversal from the persister to the normal state.

### Dew point

Because temperature and RH are significantly associated with *α*, we further examined the correlation between *α* and dew point. Table 3 also lists partial correlation results between the various dew point factors and the switch rate *α*, while controlling for solar radiation, wind speed and produce type. Dew point average, minimum (results not shown) and range followed similar trends in correlation with *α* as those same parameters for RH.

The role of free water in bacterial cell behavior and viability on leaves cannot be overstated as it serves to ensure movement, multiplication, and acquisition of soluble nutrients, to mention only these few [46–48]. Lack of water may cause osmotic stress at microsites on leaves where solutes are at high concentrations [49]. It is noteworthy that we previously reported that population sizes of *Salmonella* declined and subsequently stabilized on dry leaves of cilantro plants maintained under low RH, then increased rapidly when dew formation was triggered on the leaves [50]. These dynamics may reflect the recovery of the small surviving subpopulation from the persister state. There is evidence that high osmolarity induces persister cell formation [21]. Furthermore, transient exposure to starvation enhances formation of persister cells in *Salmonella* [51] and an active starvation stress response mediates protection of *Pseudomonas aeruginosa* against several antibiotics [52]. Both osmotic stress and starvation may result from lack of free water, which concentrates solutes on leaves and restricts substrate availability to bacteria. Hence our model’s demonstration that *Salmonella* switch rate to persister cells on leaves is negatively correlated with dew point, which reflects the potential for the presence of free water on aerial plant surfaces, is consistent with current knowledge of persister formation in this pathogen.

## Conclusions

**Fig. 3:**
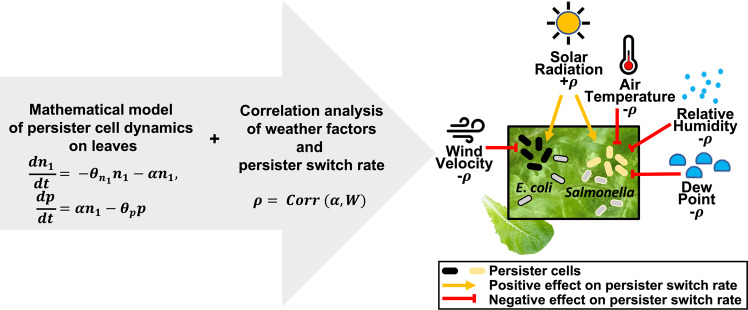
Schematic diagram illustrating mechanistic model prediction of switch rate from normal to persister cell and correlation analysis with weather factors; increased solar radiation intensity promoted the persister switch rate in both *E. coli* and *Salmonella* on leaves. On the contrary, increased wind speed inhibited the persister switch rate in *E. coli*, and increased RH, temperature, and dew point similarly had a negative effect on the persister switch rate in *Salmonella*.

The application of our persister model to *E. coli* and *Salmonella* survival data on plants from a previously published field study [9] and subsequent correlation analysis with weather parameters provide new knowledge of the physical factors that may enrich for bacterial persister cells in natural environments. Figure 3 illustrates the conclusions from our study. The strong positive association of predicted *E. coli* and *Salmonella* persister cell formation rates in the phyllosphere with solar radiation intensity accords with the reported induction of persistence by the SOS response *in vitro* in *E. coli* [33]. Likewise, the negative correlation of estimated *Salmonella* persister switch rates with weather parameters that promote water deposition on leaves is supported by previous observations that reversely, osmotic stress and starvation, both of which may be caused by low water availability in bacterial habitats, trigger the production of *Salmonella* persister cells [21, 51]. Our finding that the predicted switch rate of *E. coli* to the persister state decreased as wind velocity increased is worthy of further investigation since the role of wind speed in the bacterial ecology of plant surfaces remains unknown. Notably, the negative correlations of persister switch rate with wind and with weather factors related to water availability on surfaces were specific to *E. coli* and *Salmonella*, respectively, in this study. While the mechanisms involved in the formation of persisters must be elucidated further at the molecular level, there is evidence that they can differ among bacterial species. For example, *Salmonella* entered the persister state via specific pathways in response to inducers relevant to internalization into macrophages, while *E. coli* did not respond similarly [51]. Hence, our results corroborate that distinct pathways can be at play in persister cell formation in different organisms under various environmental pressures.

Since persister cells have enhanced survival to antimicrobial compounds, low pH, and reactive oxidative species [21], all of which underlie produce decontamination strategies, a better understanding of enteric pathogen physiology on crops allows for critical insight into the efficacy of different approaches to improve their safety for human consumption. New knowledge of the environmental factors that promote these subpopulations of dormant cells is not only of interest in public health but also more broadly informs microbial ecology. Our mechanistic model allowed for identification of weather parameters as an important measure of the predictable physiological state of a subpopulation of bacterial cells on plants. In a heterogeneous environment, such as the phyllosphere and other microbial habitats, the physiology of individual cells may determine the ultimate fate of the broader population over prolonged time scales. Bacterial persistence clearly adds to a spectrum of strategies that bacteria may employ to survive in the environment. These cells could therefore dictate the outcome of a range of environmental interventions, including microbiome engineering, bioremediation, protection against plant diseases with biocontrol agents, and antibiotic treatment, to mention only these few. Knowledge of the physicochemical factors that induce persister cells may serve as an important prediction tool. Thus, our study additionally proposes a new framework from which to investigate bacterial behavior in natural environments in the context of a problem that spans multiple scales and categories of information.

## Supplementary information

Supplementary Information

## Data Availability

All data used in this study can be found at https://github.com/abelias/die_off.
