# Supplementary material for: Weather stressors correlate with Escherichia coli and Salmonella enterica persister formation rates in the phyllosphere: a mathematical modeling study
Source: ISME Commun. 2022 Sep 27;2:91. doi: 10.1038/s43705-022-00170-z (PMC9723732; doi:10.1038/s43705-022-00170-z)
Supplement: Supplementary file 1 — Supplementary Information [file 43705_2022_170_MOESM1_ESM.docx]

**Appendix:**

**Results for Parameter fitting:**

**Table S1.** Fitting results from model equations (3a)-(3c) for fast decay ${\theta_{n}}_{1}$(1/h), slow decay $\theta_{p}$(1/h), and switch rate $\alpha$ (1/h) with respect to the average population size dynamics for respective field trials illustrated in Figure 2.

|  | ${\theta_{n}}_{1}$ (1/h) | $\theta_{p}$ (1/h) | $\alpha$ (1/h) | RMSE (log10 cfu/100g) |
| --- | --- | --- | --- | --- |
| California (LE) | 8.2e-1 | 2.2e-2 | 2.3e-4 | 1.6e-2 |
| California (LS) | 5.1e-1 | 2.7e-2 | 3.8e-3 | 3.9e-3 |
| California (SE) | 7.6e-1 | 2.7e-2 | 2.8e-4 | 1.1e-2 |
| California (SS) | 4.9e-1 | 3.7e-2 | 7.4e-3 | 5.3e-3 |
| Spain (LE) | 8.2e-1 | 2.2e-2 | 1.3e-4 | 4.5e-3 |
| Spain (LS) | 5.8e-1 | 3.9e-2 | 7.8e-4 | 9.2e-3 |
| Spain (SE) | 6.1e-1 | 6.3e-2 | 1.6e-3 | 3.7e-3 |
| Spain (SS) | 4.1e-1 | 4.6e-2 | 1.5e-3 | 1.3e-2 |
| New York (SE) | 6.8e-1 | 4.4e-2 | 2.2e-3 | 7.5e-3 |
| New York (SS) | 4.9e-1 | 3.0e-2 | 4.0e-3 | 6.1e-3 |

LE = lettuce and *E. coli*, LS = lettuce and *Salmonella*, SE = spinach and *E. coli*, SS = spinach and *Salmonella*, RMSE = root mean square error from the optimal fits for *E. coli* or *Salmonella* dynamics in each trial

**Details for parameter fitting:**

Given the biphasic nature of the data (and our model assumptions A-D), there is no ambiguity in obtaining initial ranges for the parameters $\theta_{n_{1}}$ and $\theta_{p}$. Furthermore, working from the model equations (3a)-(3c) and our assumptions we obtained bounds on $\alpha$ as follows:

*Lower bound:* Based on our assumption that dp/dt_t=0_ >0, that is, we assumed that moving from the inoculum solution to the plant surface is stressful for the bacteria and thus, persisters should start forming on the plant surface just following inoculation, from equation (3b) we have:

$$0< {\frac{dp}{dt}|}_{t=0}= \alpha n_{0}- \theta_{p}p_{0},$$

which indicates that $\alpha>\frac{\theta_{p}p_{0}}{n_{0}}$, where $p_{0}$ is the initial persister population and $n_{0}$ is the initial normal cell population. Setting $p_{0}=1$, we have, $\alpha>\frac{\theta_{p}}{n_{0}}$, for the lower bound.

*Upper bound:* Since equation (3a) decouples from equation (3b), we solved directly for $n_{1}$ and then used this to solve directly for$p$. It follows that for any time $t\geq0$, the persister population $p(t)$ can be expressed as

$$p\left( t \right)= e^{-\theta_{p}t}\left( \hat{p_{0}}+\frac{\alpha n_{0}}{\alpha+\theta_{n_{1}}-\theta_{p}} \left[ 1-e^{(-\alpha-\theta_{n_{1}}+\theta_{p})t} \right] \right)$$

Since $\theta_{n_{1}}\gg\theta_{p}$, $1-e^{\left( -\alpha-\theta_{n_{1}}+\theta_{p} \right)t}<1$, so we have that $p\left( t \right)\leq\hat{p_{0}}+\frac{\alpha n_{0}}{\alpha+\theta_{n_{1}}-\theta_{p}}$, for all $t\geq0.$

Assuming that the tail of the observed population is comprised of a majority of persisters, given our model structure, the persister population $p\left( t \right)\leq p_{0}^{*}$for any $t\geq0$, where $p_{0}^{*}$ is the intersection of the curve $ce^{-\theta_{p}t}$ (i.e. the approximate curve describing the tail of the population) and the $y$-axis ($t$=0). Thus, we insisted that for all $t\geq0, p\left( t \right)\leq\hat{p_{0}}+\frac{\alpha n_{0}}{\alpha+\theta_{n_{1}}-\theta_{p}}\leq p_{0}^{*}$. From these inequalities, it follows that: $\alpha\leq\frac{{(\theta}_{n_{1}}-\theta_{p})p_{0}^{*}}{n_{0}-p_{0}^{*}}\approx\frac{{(\theta}_{n_{1}}-\theta_{p})p_{0}^{*}}{n_{0}}$, since $n_{0}\gg p_{0}^{*}$. Thus, for our approximation of the upper bound for $\alpha$ we used $\frac{{(\theta}_{n_{1}}-\theta_{p})p_{0}^{*}}{n_{0}}$ .

These bounds allowed us to narrow the initial parameter inputs for the minimization algorithm carried out by the *fminsearch* function, leading to error minimization on the restricted parameter space. Notice that each trial to which the model was fit had seven data points (at t=0, 4, 8, 24, 48, 72, 96 h). Given this, the biphasic trend of the data, the bounds determined for possible $\alpha$ values, and the assumption on the initial persister population, together ensure the practical identifiability of $\theta_{n_{1}}$, $\theta_{p}$, and $\alpha$.

To limit bias and account for unknown bacteria population levels in a small number of field samples that were nevertheless positive by enrichment, an imputation strategy was performed by Belias et al. (2020) based on ten imputation rounds per sample. Because the distributions resulting from these imputations relative to the time points in question were almost identical, we used population data from a single imputation. That is, given a single imputation for a particular plot level across the aforementioned sample times, $y_{k}$ represents the average bacteria population at sample time $t_{k}$. Belias *et al.* (2020) provide detailed information regarding the microbial testing strategy, imputation procedure, and individual creation of plot level data subsets corresponding to each produce/bacteria pair in their study [1].

It is important to clarify that while imputation values (mainly for data collected at times past 48 h post-inoculation) were used in the fits for the model, the “tail” of the measured populations for the purpose of our study was effectively determined by the 24 and 48 h time points with respect to each field trial. In terms of the raw field data (from Belias *et al.* (2020)) for the trials we used in the current study, only one trial in Spain (lettuce - *Salmonella*) exhibited zero colonies on enumeration for the 20 samples collected at t=48 h (but had 20 out of 20 samples above the limit of detection of 6 CFU/100g at t=24 h). Only two (Spain: spinach -*Salmonella*; Spain: lettuce-*Salmonella*) of the other 20 trials (note we used a total of 21 field trials Belias *et al.* (2020)) had 40% of the 20 samples above the limit of detection at t=24 and 48 h. The rest of the trials had at least 50% of samples above the limit of detection at t=24 and 48 h. These data thus suggest that the tails of the said populations are legitimate.

**Justification for using the relationship** $\boldsymbol{\alpha}_{\boldsymbol{d}}\boldsymbol{\approx k\alpha}$**:**

In order to justify the relationship $\alpha_{d}\approx k\alpha$, we compare the model (2a)-(2d) and (3a)-(3c) outputs in the context of bi-phasic population decay. Since $\hat{p_{0}}\approx0$, using model (2a)-(2d) we solved for $\hat{p}$ for $t\geq0$, to obtain

$$\hat{p}\left( t \right)\approx\frac{-\alpha_{d}n_{10}}{\theta_{\hat{p}}-\left( \alpha_{d}+\theta_{n_{1}} \right)}e^{-\theta_{\hat{p}}t}+\frac{\alpha_{d}n_{10}}{\theta_{\hat{p}}-\left( \alpha_{d}+\theta_{n_{1}} \right)}e^{-{(\alpha_{d}+\theta}_{n_{1}})t}, (4a)$$

and using model (3a)-(3c), we solved for $p$ for $t\geq0$, to obtain

$$p\left( t \right)\approx\frac{-\alpha n_{0}}{\theta_{p}-\left( \alpha+\theta_{n_{1}} \right)}e^{-\theta_{p}t}+\frac{\alpha n_{0}}{\theta_{p}-\left( \alpha+\theta_{n_{1}} \right)}e^{-{(\alpha+\theta}_{n_{1}})t}, (4b)$$

Note that $p\left( t \right)$ from equation (4b) describes the tail of the population (coming from model (3a)-(3c)) and note that $n_{2}$(t) (coming from equation (2b)) describes the same tail. This means that for $t\geq t^{*}\approx\frac{1}{\theta_{n_{1}}}$ , $p\left( t \right)\approx n_{2}$(t). In addition, for the same time interval, our assumption on the persister population, that $\hat{p}(t)\approx kn_{2}\left( t \right)$, indicates that $\hat{p}(t)\approx kp(t)$. Comparing formulas (4a) and (4b), and using the fact that $\theta_{n_{1}}\gg\alpha_{d}$, $\theta_{n_{1}}\gg\alpha$, $\theta_{\hat{p}}$ = $\theta_{n_{2}}=\theta_{p}$, and $n_{10}\approx n_{0}$, it follows that $\alpha_{d}\approx k\alpha$.

**REFERENCE**

1. Belias AM, Sbodio A, Truchado P, Weller D, Pinzon J, Skots M, et al. Effect of weather on the die-off of *Escherichia coli* and attenuated *Salmonella enterica* serovar Typhimurium on preharvest leafy greens following irrigation with contaminated water. Appl Environ Microbiol. 2020; 86:e00899-20.
